# Editorial: Advancing immunotherapy in the elderly: overcoming metabolic and inflammatory barriers

**DOI:** 10.3389/fimmu.2025.1716995

**Published:** 2025-10-10

**Authors:** Zechen Wang, Pin Guo, Chao Liu, Guang-Liang Chen

**Affiliations:** ^1^ Department of Neurosurgery, The Affiliated Hospital of Qingdao University, Qingdao, China; ^2^ Department of Immunology, Harvard Medical School, Boston, MA, United States; ^3^ Department of Cancer Immunology & Virology, Dana-Farber Cancer Institute, Boston, MA, United States; ^4^ Department of Medical Oncology, Fudan University Shanghai Cancer Center; Department of Oncology, Shanghai Medical College, Fudan University, Shanghai, China

**Keywords:** geriatric oncology, immunosenescence, ICIS, immunometabolism, inflammaging, cancer cachexia, lncRNA, aging

As populations age, cancer care is being reshaped by a growing burden of malignancy in older adults (1). Aging is the dominant risk factor for cancer and brings a distinct host biology, including immunosenescence, inflammaging, and dysregulated immunometabolism, often compounded by comorbidity and frailty (2–4). Together, these forces alter treatment selection, tolerance, and outcomes. This Research Topic, *Advancing Immunotherapy in the Elderly: Overcoming Metabolic and Inflammatory Barriers*, assembles studies that directly address these challenges. The contributions move beyond treating age as a covariate to interrogate the aging host biology and offer strategies to sharpen diagnostics, improve therapeutic efficacy, and mitigate debilitating systemic symptoms.

## Redefining the efficacy of immunotherapy in the aging host

A prevailing concern in geriatric oncology has been that immunosenescence may render immune checkpoint inhibitors (ICIs) ineffective in older patients. The work presented in this topic provides a powerful rebuttal to this dogma. A multicenter observational analysis by Drobniak et al. evaluated the combination of nivolumab and ipilimumab in older adults with metastatic renal cell carcinoma (mRCC). Their findings provide reassuring real-world evidence: patients aged ≥65 years demonstrated superior outcomes compared to younger patients (<65 years), with longer progression-free survival, higher ORR/DCR, and acceptable immune-related toxicity profiles. This challenges the conventional wisdom that age alone should be a limiting factor for immunotherapy, proving that an “aged” immune system remains remarkably capable of being harnessed against cancer.

Building on optimization of the host-tumor interface, Zhan et al. explored a multimodal strategy in a real-world cohort with locally advanced gastric cancer (LAGC). By integrating ICIs into a neoadjuvant regimen with chemotherapy and anti-angiogenic therapy, they achieved impressive rates of pathological complete response (23.7%) and major pathological response (47.4%), with R0 resection 97.4% and encouraging 1-year overall survival (OS) 100%/disease-free survival (DFS) 94.7%. Median OS and DFS had not been reached at the time of reporting. Importantly, while not elderly-specific, the cohort’s median age was 65 and 63.2% were >60 years old, supporting applicability to many older candidates for surgery. These data illustrate a practical multimodal path to enhance surgical readiness and early outcomes; longer follow-up is needed to confirm durability. When considered alongside real-world mRCC results showing at least comparable disease control and PFS in patients ≥65 on nivolumab plus ipilimumab, the case for expanding ICI use in well-selected older adults, and for combination strategies that optimize the host-tumor interface, grows stronger.

## Targeting the inflammatory milieu to improve patient outcomes

Beyond direct anti-tumor activity, a critical frontier in geriatric oncology is managing the systemic consequences of cancer, which are often exacerbated by inflammaging. Cancer cachexia, a debilitating syndrome of weight loss and muscle wasting driven by systemic inflammation, severely impacts quality of life and treatment tolerance. The work of Chen et al. provides a targeted therapeutic approach. They investigated the use of tocilizumab, an IL-6 receptor inhibitor, in combination with corticosteroids to manage cancer cachexia. Their results show that this immunomodulatory strategy was associated with improved inflammatory and nutritional indices, weight gain, increased strength, and higher rates of restarting anticancer therapy, without excess infections or ICU admissions. These findings support targeted anti-inflammatory approaches that directly block a key cytokine pathway as an effective bridge to maintain or resume cancer treatment.

## Innovating diagnostics for a new era of precision medicine

Effective treatment begins with accurate and early detection; a need that is particularly acute in frail elderly patients for whom invasive procedures carry higher risk. In this Topic, the study on exosome-derived lncRNA PITPNA-AS1 in pleural effusions by Chen et al. shows how a liquid-biopsy signal can aid differential diagnosis across lung cancer subtypes and track disease burden. Their research shows that exosomal lncRNA PITPNA-AS1 distinguishes malignant from benign lung conditions with high sensitivity and specificity across several lung-cancer subtypes. Mechanistically, PITPNA-AS1 binds FMR1, blocks its ubiquitination, and thereby stabilizes the protein, an interaction that may drive oncogenesis. Consequently, the study moves beyond diagnostics: by implicating PITPNA-AS1 in tumorigenesis, it reveals a potential therapeutic target at the nexus of cellular metabolism and cancer signaling. Overall, PITPNA-AS1 emerges as a promising, non-invasive biomarker; future work should assess its prognostic value and longitudinal performance.

## Conclusion and future outlook

Collectively, the studies in this Topic show that we can expand the appropriate use of ICIs in older adults, modulate inflammatory and angiogenic barriers to improve perioperative efficacy, and deploy non-invasive diagnostics that ease the burden on frail patients. The path forward is not to withhold therapy based on chronological age, but to personalize care by integrating immunosenescence, inflammaging, and immunometabolic considerations, so that more older patients can both receive and benefit from cancer immunotherapy.
